# Osteocytes in the Metastatic Bone Niche: Mechanistic Pathways and Therapeutic Targets

**DOI:** 10.3390/ph19040644

**Published:** 2026-04-20

**Authors:** Alhomam Dabaliz, Mohamad Bakir, Lana Fatash, Mais Aldoush, Khalid Said Mohammad

**Affiliations:** 1 Department of Clinical Skills, College of Medicine, Alfaisal University, Riyadh 11533, Saudi Arabia; almdabaliz@alfaisal.edu; 2 Department of Medicine, College of Medicine, Alfaisal University, Riyadh 11533, Saudi Arabia; mbakir@alfaisal.edu (M.B.); lfatash@alfaisal.edu (L.F.); maldoush@alfaisal.edu (M.A.); 3 Department of Anatomy, College of Medicine, Alfaisal University, Riyadh 11533, Saudi Arabia

**Keywords:** osteocytes, bone metastasis, metastatic niche, mechanotransduction, sclerostin (SOST), targeted therapy

## Abstract

Osteocytes, once viewed mainly as passive bone-embedded cells, are now recognized as active regulators of the metastatic bone niche. Emerging evidence indicates that these cells integrate mechanical, inflammatory, and tumor-derived cues to influence metastatic seeding, dormancy, reactivation, and lesion progression in bone. This review synthesizes current understanding of osteocyte contributions to skeletal metastasis. We discuss core signaling axes, including osteocyte-derived RANKL/OPG balance, Wnt antagonists (sclerostin/DKK1), mechanotransduction pathways (Piezo1 signaling and connexin-43 hemichannels), and osteocyte paracrine mediators (extracellular vesicles and senescence-associated factors), and examine how each axis modulates tumor cell dormancy, osteolysis, or osteoblastic progression. We then review translational strategies targeting osteocytes, recent preclinical and clinical insights. Emerging biomarkers (e.g., serum sclerostin, DKK1, bone turnover markers) and immune–skeletal imaging approaches are also considered. Controversies, including the paradoxical effects of sclerostin blockade and the identity of in vivo RANKL sources, are discussed. Finally, we outline key knowledge gaps and propose endpoints for future trials. In summary, an osteocyte-centric perspective reveals novel targets and strategies for managing bone metastases, guiding future translational research.

## 1. Introduction

Traditionally, bone biology research took an osteoclast-centric perspective of bone resorption and metastasis, with osteoclasts being believed to be the main offenders of the skeletal destruction in metastatic disease. This osteoclast-focused “vicious cycle” model explained the interaction between tumor cells and osteoclasts. It suggested that tumor cells increase the differentiation and activity of osteoclasts, which results in bone degradation and the release of growth factors from the matrix that, in turn, promote tumor growth, thus providing a clear mechanistic link between resorption and metastatic progression [1]. However, this view does not fully capture the cellular complexity of the metastatic bone microenvironment.

Osteocytes, the most abundant cells in bone and the principal cells embedded within the mineralized matrix, are increasingly recognized as active organizers of bone homeostasis rather than passive bystanders [2]. Because of their unique anatomical position and extensive communication network within bone, osteocytes are well placed to integrate local and systemic cues and to coordinate interactions among tumor cells, osteoblast-lineage cells, osteoclasts, stromal cells, and immune components of the bone niche [2,3]. Emerging evidence suggests that osteocytes influence multiple stages of metastatic progression, from early niche conditioning to later remodeling of established lesions, thereby shaping both tumor behavior and skeletal response.

**Figure 1:**
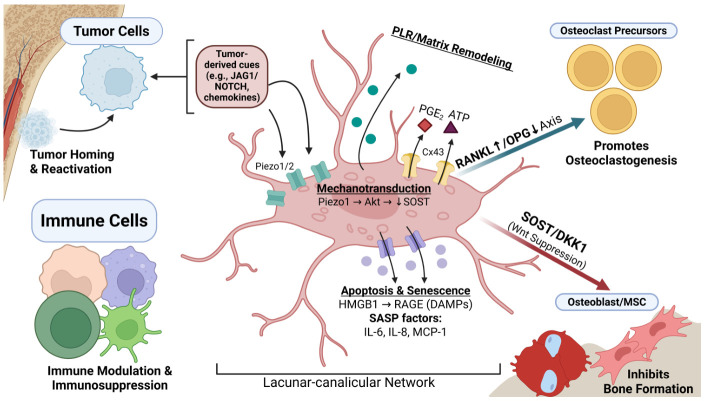
Osteocytes embedded within the lacunar–canalicular network integrate tumor-derived cues, mechanical loading, and immune-associated signals. These inputs converge on major osteocyte effector programs, including mechanotransduction through Piezo1/2 and connexin-43 hemichannels, release of ATP and PGE2, regulation of osteoclastogenesis through the RANKL/OPG axis, suppression of osteoblast/MSC activity through SOST/DKK1-mediated Wnt inhibition, apoptosis/senescence-associated inflammatory signaling, and perilacunar/canalicular remodeling (PLR). Together, these osteocyte-driven pathways shape immune modulation, tumor cell seeding/outgrowth, and osteolytic or mixed remodeling of metastatic bone. Created in BioRender. Mohammad, K. (2026) https://BioRender.com/azoaj5o (accessed on the 12 of April 2026).

This review argues that osteocytes are not passive bystanders in metastatic bone disease but integrative regulators of niche state. We therefore organize the literature around how osteocytes influence metastatic stages, the major signaling axes through which they act, and the translational implications of targeting osteocyte-driven remodeling, mechanosensation, senescence, and immune crosstalk. Figure 1 summarizes the major signaling axes involved in octeocyte-tumor interactions.

## 2. Osteocyte Biology

Osteocytes are the mature bone cells that reside in the mineralized matrix. They communicate with each other through a highly organized lacunocanalicular network (LCN) that also facilitates nutrient exchange and enables interaction with the surrounding extracellular matrix [4]. It is a misconception to think that osteocytes are merely metabolically inactive cells; as they actually have the capability to alter their immediate surroundings through perilacunar/canalicular remodeling (PLR), which is a key factor for the quality and mechanical properties of bone [4]. Osteocytes achieve that by synthesizing enzymes that facilitate both matrix degradation and renewal. Among the secreted enzymes that break down collagen and other organic materials are matrix metalloprotease 13 (MMP13) and cathepsin K (CTSK), while carbonic anhydrase 2 (CA2) helps create an acidic environment in the perilacunar area, thus allowing mineral dissolution. If such enzymatic processes do not function properly, this leads to the disintegration of the LCN, alterations in bone material properties, and increased bone fragility; therefore, osteocytes are key to matrix turnover and skeletal homeostasis [4].

Beyond their role in maintaining and remodeling mineralized bone, osteocytes act as endocrine and paracrine regulators that coordinate bone remodeling and systemic mineral metabolism [5]. Through the RANKL/Osteoprotegrin (OPG) pathway, osteocytes regulate osteoclast formation and bone resorption. Additionally, they precisely regulate bone formation by the release of sclerostin (SOST) and Dickkopf-1 (DKK1), which are very efficacious inhibitors of Wnt/β-catenin signaling [6]. Furthermore, osteocytes produce several signaling molecules, including interleukin 6 (IL-6), ATP, prostaglandin E_2_ (PGE_2_), and CXCL chemokines, which, in addition to modulating osteoblast–osteoclast coupling, also regulate immune–bone interactions in the skeletal microenvironment. Consequently, osteocytes become major regulators of mineral balance, both locally in the bones and at the whole-body level [5].

A significant aspect of PLR and osteocyte signaling is the ability of osteocytes to sense and respond to mechanical forces. Osteocytes act as the primary mechanosensors within bone [7]. Mechanical loading results in fluid shear stress within the LCN that is detected by mechanosensitive pathways with Piezo1 and Piezo2 ion channels’ involvement, leading to calcium influx and the activation of downstream anabolic signaling pathways [7]. Furthermore, mechanical stimulation leads to connexin-43 (Cx43) hemichannels’ opening, which in turn releases ATP and PGE_2_, leading to amplification of mechanotransduction as well as bone remodeling [7]. These mechanical forces are integrated into the transcriptional factors YAP and TAZ, which translocate into the nucleus on loading to modulate gene expressions linked to survival signaling, differentiation, and bone formation of osteocytes. Essentially, these mechanosensory inputs endow osteocytes with the capability to transduce these mechanical forces into bioactive signals that are fundamental to the skeletal strength maintenance [7].

## 3. Osteocytes Across the Metastatic Cascade

**Figure 2:**
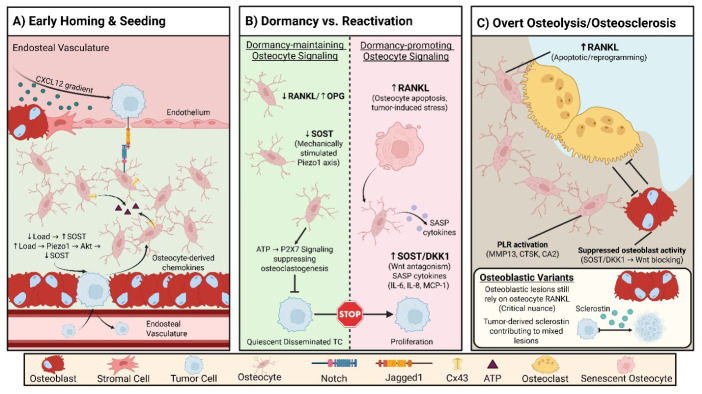
Stage-specific roles of osteocytes across the metastatic cascade in bone. (**A**) Early homing and seeding. Tumor cells extravasate near the endosteal vasculature and are retained by chemokine gradients (classically CXCL12-driven), while osteocyte mechanosensing (loading-dependent regulation of SOST via Piezo1→Akt) and osteocyte-derived chemokines shape early niche architecture and permissiveness. (**B**) Dormancy versus reactivation. Osteocytes can support dormancy-maintaining conditions through reduced pro-resorptive signaling (lower RANKL relative to OPG), suppression of Wnt antagonism (lower SOST), and ATP–purinergic signaling (e.g., ATP→P2X7) that restrains osteoclastogenesis. In contrast, tumor stress and osteocyte apoptosis/senescence can increase RANKL and SASP cytokines (e.g., IL-6, IL-8, MCP-1) and elevate Wnt antagonists (SOST/DKK1), favoring osteoclast activation and DTC outgrowth. (**C**) Overt osteolysis/osteosclerosis. During established lesions, osteocyte reprogramming and apoptosis promote osteoclastogenesis through increased RANKL, while PLR enzyme activation (MMP13, CTSK, CA2) contributes to local matrix remodeling and amplifies osteolytic progression. Concurrent osteocyte-derived Wnt inhibition (SOST/DKK1) suppresses osteoblast activity; osteoblastic/mixed variants may still rely on osteocyte-driven RANKL-dependent remodeling, with tumor-derived sclerostin proposed as a contributor to mixed phenotypes in some contexts. Created in BioRender. Mohammad, K. (2026) https://BioRender.com/0xt8y05 (accessed on the 12 of April 2026).

Osteocytes influence the metastatic process at multiple temporal stages, from early niche conditioning to late-stage remodeling and skeletal-related events. Framing osteocyte functions along the metastatic cascade helps distinguish stage-specific mechanisms (e.g., dormancy maintenance versus osteoclast-driven reactivation) and clarifies which therapeutic strategies are most plausible for prevention, containment, or treatment of established lesions. This cascade-based structure also provides a rationale for combining standard antiresorptives with osteocyte-targeted interventions and for embedding osteocyte-centric biomarkers into clinical trial endpoints (Figure 2).

### 3.1. Homing, Seeding, and Early Colonization

At the beginning of bone metastasis, tumor cells that spread reach the bone marrow and the endosteal niche, where they are trafficked and retained by chemokine gradients, especially the CXCL12–CXCR4 axis, thus mimicking hematopoietic stem cell homing [8]. Whilst osteoblasts and stromal cells constitute the main sources of CXCL12 in this niche, osteocytes are gaining recognition as a significant part of the endosteal microenvironment that influences chemokine fields indirectly through the control of bone remodeling and niche architecture [1] (Figure 2A). There is increasing evidence that osteocytes respond to tumor-induced changes by altering their secretory and signaling profiles. In this way, they affect tumor cell survival, dormancy, and the first colonization of tumor cells in the bone marrow [1]. During this phase of tumor-bone interaction, there is also activation of the Notch signaling pathway via tumor-bone cell crosstalk. Specifically, tumor-expressed Jagged1 (JAG1) binds Notch receptors on bone-resident cells, thus promoting a pro-metastatic niche with increased osteoclastogenic and inflammatory signaling [9]. While it has been a common theme in most mechanistic studies to limit their focus to osteoblasts and stromal cells, recent studies have concluded that dysregulated Notch signaling can induce osteocyte apoptosis and thus disrupt bone homeostasis. This finding strengthens the idea that osteocytic Notch activation may be a factor in remodeling the early metastatic niche [10].

### 3.2. Dormancy vs. Reactivation

Osteocytes play a role in maintaining the equilibrium between dormancy and reactivation of tumor cells that have spread to bone by influencing the production of factors that lead to osteoclast formation and the bone remodeling environment where dormant tumor cells (DTCs) are located [11]. They express both RANKL and OPG; therefore, they are capable of influencing the RANKL/OPG ratio, favoring increased RANKL to promote osteoclast formation and bone resorption, which has been linked to dormancy escape of DTCs [11]. Osteocytes produce other molecules that antagonize Wnt signaling, such as sclerostin and DKK1m, which have been shown to inhibit osteoblast activity and alter the bone microenvironment. Modulation of these Wnt antagonists may affect the niche, favoring maintenance of dormancy or escape [11]. Different mechanical inputs are detected by osteocytes, which then regulate the secretion of signaling molecules that influence the activities of both osteoblasts and osteoclasts. Additionally, changes in mechanical signaling have been associated with modifications in the RANKL/OPG system and their consequent impact on the bone remodeling niche [12]. Physiological mechanical loading increases the opening of Cx43 hemichannels in osteocytes and the extracellular release of ATP, which can decrease osteoclast activity, thereby helping maintain a more quiescent niche. On the other hand, a loss of these signals can contribute to osteoclast activation and DTC reactivation [11]. When combined, these signals that sustain bone cells (changes in RANKL/OPG, alterations of Wnt antagonists, and mechanically regulated ATP release through Cx43) essentially form a live microenvironment that may limit DTCs to dormancy or facilitate their reactivation and progression to dissemination in bone (Figure 2B).

### 3.3. Progression to Osteolysis and Osteosclerosis

As bone metastasis progresses to form osteolytic lesions, a rise in osteocyte apoptosis seems to be localized with the increased expression of osteoclastogenic signals such as RANKL, which not only attracts osteoclast precursors but also promotes their differentiation and resorptive activity, thus increasing the well-known “vicious cycle” of bone destruction that tumor growth in bone feeds on [1]. By releasing RANKL, osteocytes can control osteoclastogenesis as well as change the structure of their surrounding perilacunar and pericanalicular matrix, as well, which is a process similar to that of osteoclastic resorption in that it requires acidification and the use of matrix-degrading enzymes such as MMP13 and CTSK, which are capable of releasing more matrix components and thus contributing to bone loss [1,13]. In metastatic microenvironments, there is an increase in Wnt antagonists from osteocytes, such as sclerostin and DKK1 that inhibit osteoblast differentiation, tipping the balance towards bone resorption over formation, therefore, synergizing with tumor-secreted factors to aggravate osteolysis [1,14]. Osteocyte apoptosis, together with the action of perilacunar bone-resorbing enzymes such as MMP13 and CTSK, and the upregulation of sclerostin and DKK1, propels the microenvironment away from new-bone formation towards resorption, thereby directly linking osteocyte function to the establishment of osteolytic lesions and the therapeutic strategy in anti-RANKL treatment (Figure 2C).

## 4. Mechanistic Axes: Osteocytes Interface with Tumor and Stroma

### 4.1. RANKL/OPG Control by Osteocytes

The role of osteocytes on RANKL/OPG balance has been the subject of much interest. Much of the literature points towards the possibility of osteocytes being a major source of RANKL in the bone environment [15]; however, a recent paper published in 2024 has demonstrated the possibility that osteocytes act as a major producer of OPG, while different osteoprogenitor cells are the major producers of RANKL [16]. The initial belief came from studies on knockout mouse models, such as the *Dmp1-cre;Tnfsf11^fl/fl^* and *Sost-cre;Tnfsf11^fl/fl^* models, where *Tnfsf11* is the gene encoding for RANKL [15,17]. The results of these studies collectively pointed to osteocytes as the major source of RANKL, as it was believed that only osteocytes were targeted by both transgenes [15]. However, multiple studies since then have revealed that other non-osteocytic cell populations are targeted by the *Dmp1* and *Sost* promoters, calling into question the validity of the prior conclusions [18,19,20]. El-Masri et al. argue that it is, in fact, osteoprogenitor cells, not osteocytes, that were the main target in these previous knockout studies, and they supported this with scRNAseq data from enriched bone marrow stroma isolated from mice. This data showed that the most abundant RANKL+ cells also expressed high levels of MMP13, a marker that is highly expressed on osteoprogenitor cells on bone surfaces [16,21]. This balance between RANKL and OPG is disrupted in the presence of denosumab, a RANKL-antibody used to reduce fracture risk and increase bone mass [22]. Studies on patients treated with denosumab have shown an elevated RANKL/OPG ratio in their blood [19,23], with a drop in OPG production that may be due to loss of surface osteoblasts that are later converted into osteocytes, 2 major sources of OPG production [16]. This elevated ratio may explain why many of these patients develop accelerated bone loss on denosumab discontinuation [24]. This phenomenon may also be relevant to increased metastatic risk, as many studies have shown that osteolysis is a major promoter of bone metastasis [25].

Taken together, the current evidence no longer supports the unqualified statement that osteocytes are the sole or universal dominant source of RANKL in vivo. Rather, osteocytes remain central regulators of the RANKL/OPG axis and may be particularly important for OPG production. In contrast, osteoprogenitor populations may account for a substantial fraction of RANKL in some settings. The translational implication is that therapeutic strategies should focus on the functional balance of the axis and the disease context, rather than assuming a single cellular source across all models of metastatic bone disease

### 4.2. Mechanotransduction

Physical exercise has been theorized to have a protective effect against cancer progression and spread. In bones, the most abundant mechanosensitive ion channel is known as Piezo1. Recent studies have explored the effects of Piezo1 stimulation on bone metastasis. Multiple studies used low-magnitude, high-frequency (LMHF) vibration as an alternative to exercise to assess its effects on bone metastasis and to compare them with those of Yoda1, a chemical Piezo1 activator. These studies revealed that the combination of LMHF and Yoda1 treatment significantly suppresses the effect on osteoclastogenesis and breast cancer cells’ extravasation and migration, as well as has a positive effect on nuclear translocation of YAP in osteocytes [26,27]. Another demonstrated effect of Piezo1 stimulation is the phosphorylation and activation of Akt, which suppresses SOST expression and reduces sclerostin, a crucial inhibitor of cancer metastasis [28]. Connexin channels are another class of ion channels that play a crucial role in cell communication. Connexin 43 (Cx43) is the most abundant and studied connexin in bone tissue [29]. Cx43 forms hemichannels that, when mechanically stimulated, serve as a direct portal for releasing prostaglandin E_2_ (PGE_2_) as well as ATP, as mentioned prior [30] (Figure 1). The released PGE2 increases β-catenin expression and decreases sclerostin expression within osteocytes, simultaneously inducing osteoblast activity while reducing osteoclast activity [30,31]. On the other hand, released ATP assists in killing tumor cells by activating the purinergic receptor P2X7 on dendritic cells, thereby activating the inflammasome pathway [32]. Recent preclinical studies have also found that antibodies targeting Cx43 and activating these hemichannels in osteocytes reduce cell growth in breast cancer and osteosarcoma, as well as improve survival rates, making them a potential therapeutic strategy for preventing cancer spread [33].

### 4.3. Cell-Fate Programs

Osteocytes can influence bone cells even after death, as apoptotic osteocytes have been shown to affect osteoclastogenesis significantly. These osteocytes show an increased secretion of RANKL, TNF-α, IL-6, VEGF, and ICAMs, among other osteoclastogenic factors. All of these factors can promote osteoclast differentiation and maturation in both direct and indirect ways [34]. Similarly, necrotic osteocytes release damage-associated molecular patterns (DAMPs) that bind to pattern recognition receptors on immune cells like dendritic cells, leading to the production of pro-inflammatory cytokines that further induce the expression of RANKL, which in turn leads to the promotion of osteoclastogenesis [34,35]. Additionally, a recent study on mice has shown that osteocytes in bones affected by breast cancer are induced into a premature form of senescence with a unique senescence-associated secretory profile (SASP) that favors osteoclastogenesis [36]. These senescent osteocytes become a major source of factors such as RANKL, MMP13, and IL-6, all of which amplify lytic potential in the bone and lead to cancer-induced bone loss [36].

### 4.4. Osteocyte-Derived Extracellular Vesicles

The role of extracellular vesicles (EVs) in intercellular communication has garnered much attention in the literature. Among bone cells, osteocytes have been most associated with EV secretion in cases of mechanical stimulation [37]. These mechanically stimulated osteocytes release EVs that carry cargo like RANKL, OPG, sclerostin, and different microRNAs (miRNAs) that have been shown to promote bone formation as well as play a role in cancer metastasis to the bone [38]. However, the evidence remains sparse, and this field requires further exploration.

### 4.5. Tumor-Driven Reprogramming of Osteocytes

Just as osteocytes have a large influence on the bone microenvironment, osteocytes themselves can be influenced by tumor-derived factors. One very significant factor is the transmembrane receptor NOTCH, which plays a pivotal role in cell differentiation and function, and is constitutively active in certain types of cancer, like breast cancer and multiple myeloma [39,40], as well as promotes tumor development and skeletal metastasis in cancers like breast and prostate cancer [41]. Jagged1 (JAG1), a major ligand of NOTCH, is expressed abundantly on osteocytes, revealing a crucial mechanism for interaction between osteocytes and tumor cells expressing NOTCH receptors [42]. The activation of osteocytes through JAG1-NOTCH interactions increases the production of RANKL, resulting in osteoclast differentiation and increased osteolysis [43]. A recent study examined melanoma cells’ ability to convert osteocytes into pro-tumorigenic cells. In this study, osteocytes exposed to conditioned media from melanoma cells exhibited high CXCL5 expression, a chemokine that promotes melanoma cell migration and invasion [44]. Such a mechanism may be present in other types of cancer and warrants further investigation.

## 5. Osteocytes and Immuno-Skeletal Crosstalk

The skeletal and immune systems interact in many ways, with recent studies showing that osteocytes play a major role in these interactions.

### 5.1. Osteoimmunology: Definition and Scope

The term osteoimmunology has been coined by Dr. Yongwon Choi to describe the science that studies the interactions between the immune system and bone and its influence on disease [45]. The cells at the center of interest for this science have traditionally been osteoblasts and osteoclasts, as their interactions with the immune system have been studied quite extensively [46], while the role of osteocytes, the most abundant and widespread cells in bone, in influencing the immune system has remained elusive for a long time [46]. However, the role of osteocytes as an immunoregulatory node has been attracting more attention recently as more interactions between osteocytes and immune processes are being explored, from the secretion of cytokines like TNF-α and RANKL, to the alteration of myeloid cell differentiation [47,48]. The unique position of osteocytes between the bone matrix, stromal cells, and immune cells, both figuratively and literally, makes them a key point of interest in the context of bone metastasis. As the immune system’s influence as a key regulator of tumor spread becomes more apparent, the importance of immunoregulatory nodes such as osteocytes and their role in cancer spread becomes clearer.

### 5.2. Osteocytes in Immune Crosstalk in Metastasis

Many features of osteocytes make them a centerpiece in the crosstalk between the immune system and bone metastasis (Figure 1). Osteocytes are not only the most abundant cells in bone, but the lacunocanalicular network they inhabit pervades the bone and forms a wide network for these cells to interact [49]. They serve a variety of roles in different processes within the bone, from mechanosensation to inflammation, and produce factors that influence these processes, like cytokines and chemokines [44,50,51,52]. Additionally, their ability to influence the recruitment and differentiation of immune cells allows them to alter the interactions between the immune system and bone metastasis, both in the bone marrow and endosteal spaces [47,53,54].

Among the changes osteocytes induce in immune cells are alterations in myeloid cell composition and differentiation [47]. One study revealed that mice with reduced osteocytes have an altered myeloid lineage differentiation with expanded myeloid progenitors, neutrophils, and monocytes, and impaired lymphopoiesis, especially B cells [47]. These changes were attributed to the acquisition of SASP in both osteogenic and myeloid lineage cells [47]. Another type of immune cell affected by osteocytes is the antigen-presenting cell, such as a dendritic cell or macrophage. These cells’ activities are induced and amplified by the increase in extracellular ATP triggered by mechanical stimulation of Cx43 on osteocytes, which activates the P2X7 receptor on antigen-presenting cells [33]. These effects ultimately facilitate the recruitment and activation of CD8+ T cells and reduce the differentiation of CD4+ T cells into regulatory T cells (Treg). This elevation in extracellular ATP can also promote tissue-resident memory T cells (CD8+ Trm), which have been shown to correlate positively with tumor growth in both mouse models and human patients [55,56,57]. These various mechanisms of interaction serve to either amplify or dampen the immune system’s function within bone, either supporting anti-tumor immunity or contributing to the formation of immunosuppressive niche states. The major osteocyte signaling pathways implicated in metastatic progression and their effects on the bone niche are summarized in Table 1.

## 6. Clinical and Translational Targets

### 6.1. Denosumab (Anti-RANKL)

Therapies for metastatic bone disease, such as bisphosphonates and denosumab, target the bone microenvironment to prevent skeletal-related events (SREs) [59]. In a systematic review based on 4 randomized clinical trials, denosumab has demonstrated a consistent ability to delay the first SRE as well as alleviate bone pain in a manner similar to zoledronic acid [60]. However, risks such as hypocalcemia and osteonecrosis of the jaw are higher with denosumab, and cost-effectiveness may favor zoledronic acid in some settings [61,62]. Extended dosing intervals for denosumab in early phase II trials show promise in reducing toxicities and costs but lack extensive supporting data, as clinical endpoints may not always correlate with outcomes [63].

Mechanistically, RANKL inhibition by denosumab prevents skeletal events, yet its impact on osteocyte-driven tumor dormancy and persistence is poorly understood [64,65]. Osteocytes regulate bone remodeling via the RANKL–OPG pathway, and experimental data suggest metastatic breast cancer cells disrupt osteocyte viability and mechanotransduction, including RANKL/OPG dysregulation [66]. Osteocyte apoptosis, induced by tumor-derived factors like BIGH3/TGFBI, impairs network communication by reducing Cx43, contributing to bone breakdown independent of osteoclast activity [67].

Dormant tumor cells (DTCs) in the bone marrow persist under quiescent conditions, with their detection serving as an early marker of metastasis and prognostic indicator [68]. The bone microenvironment plays a critical role in metastasis dynamics, with osteoblast activity promoting dormancy and osteoclast-driven bone resorption reactivating DTCs through TGF-β, Wnt, and Notch pathways [65,69]. While denosumab targets RANKL signaling, its influence on osteocyte apoptosis and the dormancy niche remains unclear, highlighting a key research gap [64,66,69].

### 6.2. Sclerostin and Wnt Modulation

Romosozumab, a monoclonal antibody targeting sclerostin, enhances bone formation and reduces resorption via activation of the Wnt signaling pathway, making it a candidate therapy for bone diseases associated with malignancies that require validation [70,71]. Its primary osteoanabolic effects have drawn attention for treating bone loss and weakness in cancer; however, concerns arise regarding systemic Wnt/β-catenin pathway activation, which is frequently dysregulated in tumors and may influence tumor–bone interactions [70,72]. Preclinical evidence suggests that in Wnt-responsive tumors, altered osteocyte signaling via Wnt activation could either promote tumor adaptation within the bone or facilitate metastasis [70,72]. This underscores the need for further studies to evaluate the safety and efficacy of anti-sclerostin therapies in metastatic bone disease, particularly in combination with anti-resorptives like bisphosphonates or denosumab, to optimize outcomes [70,71,72].

DKK1 inhibitors, such as BHQ880, offer a complementary approach to treating cancers like multiple myeloma, in which bone disease is characterized by increased osteoclast activity and suppressed osteoblast function [73,74]. DKK1-mediated Wnt inhibition plays a central role in osteoblast suppression and residual bone destruction, making anti-DKK1 strategies a rational choice for addressing the mixed osteolytic and osteoblastic states seen in myeloma bone disease [73,74,75]. By reversing Wnt pathway impairment, anti-DKK1 agents promote bone formation and repair while disrupting the vicious cycle of tumor–bone marrow niche interactions that drive both bone destruction and tumor progression. However, questions remain about how bone formation alone influences disease behavior and how to integrate anti-DKK1 therapies into standard regimens alongside anti-resorptives [74,76,77]. Further research is necessary to understand their impact on the tumor–bone interface and optimize their clinical application [73,74,75].

### 6.3. Mechanotherapy and Mechanosensors

#### 6.3.1. Pizeo1 Agonism and LMHF Vibration

Piezo1, a key mechanosensor in bone physiology, plays an essential role in osteocyte mechanotransduction by converting mechanical signals into biochemical responses critical for bone remodeling and homeostasis [50,78,79]. Recent proof-of-concept preclinical studies suggest that enhancing osteocyte mechanosensation via Piezo1 agonism, such as the tool compound Yoda1, combined with low-magnitude, high-frequency (LMHF) vibration, can restrain metastatic behavior in bone-related cancers. For example, Yoda1 and LMHF vibration have been shown to regulate osteocyte signaling, reducing MDA-MB-231 breast cancer cell migration and extravasation, which are critical steps in metastasis [26,27]. Preclinical data also suggest that vibration-based interventions can influence the metastatic bone microenvironment, with in vivo studies demonstrating reduced breast cancer bone metastasis and vascularization [80].

The translational roadmap for Piezo1-based therapies includes developing safer pharmacological modulators and personalized mechanical loading prescriptions, such as LMHF vibration, to enhance osteocyte mechanosensation without adverse effects [81,82,83,84,85]. While not yet clinically established, these approaches hold promise not only for preventing bone loss in mechanical unloading or age-related osteoporosis but also for mitigating metastatic progression by targeting the bone microenvironment [26,27,80,86,87].

#### 6.3.2. Cx43 Hemichannel Activation

Cx43 hemichannels are critical mediators of osteocyte communication and play a key role in bone remodeling and metastatic progression. Activation of Cx43 hemichannels using targeted peptides (e.g., Cx43-M1/M2) or antibodies has emerged as a novel osteocyte-centric therapy class with the potential to curb bone metastases and warrants further clinical testing. Preclinical animal studies using monoclonal Cx43-M2 antibodies have shown an effective ability to inhibit the growth of breast cancer in mouse breast carcinoma in wild-type mice, as well as human breast cancer in immunocompromised mice in vivo [33]. These strategies reduce osteocyte apoptosis, suppress osteoclast activity, and promote osteoblast-mediated bone formation, helping to maintain bone integrity in metastatic disease contexts. By restoring osteocyte functionality and reducing pathological bone resorption, Cx43-based therapies may also prevent the vicious cycle of tumor–bone interactions, where bone-derived factors promote tumor survival and progression [87]. Further research is needed to refine the application of Cx43-targeted interventions, including their integration with Piezo1-based therapies and anti-resorptive agents, as part of a comprehensive strategy to address bone loss and metastasis in cancer [78,86,87].

### 6.4. PLR Enzymes and Acidification

Osteocytes play an active role in bone microenvironment remodeling through perilacunar remodeling (PLR), a process that involves localized matrix degradation and mineral mobilization. This capability becomes particularly relevant in cancer-associated bone diseases, where tumors can exploit osteocyte-driven remodeling to support skeletal colonization and growth [88,89]. Osteocytes employ molecular pathways overlapping with classical bone resorption mechanisms, including enzymes such as MMP13, CTSK, and CA2, which drive PLR and acidification of the lacunar microenvironment [90,91]. Acidification is a central mechanism enabling osteocytes to dissolve mineral components and modify matrix biochemistry, thereby influencing cancer cell behavior within the bone niche [91,92].

Targeting PLR-associated enzymes like MMP13, CTSK, and CA2 offers a therapeutic approach to blunt osteocyte-driven matrix mobilization near tumor foci. However, systemic inhibition of these pathways raises safety concerns, such as the increased stroke risk observed in preclinical animal trials with the cathepsin K inhibitor odanacatib, emphasizing the need for local or cell-targeted strategies [93]. Localized modulation of PLR could limit tumor-favorable remodeling while reducing systemic adverse effects, particularly in metastatic bone disease, where osteocyte activity contributes to the permissive microenvironment for tumor progression [88,90,91].

Future therapeutic efforts should focus on targeted delivery of inhibitors to osteocytes or the tumor-bone interface to mitigate osteocyte-driven matrix degradation while minimizing systemic risks. This approach aligns with the emerging recognition of osteocyte PLR as a clinically relevant contributor to cancer-induced bone remodeling, offering a path to reduce osteocyte-mediated tumor progression in the bone microenvironment [88,89,90,91,92,94].

### 6.5. Purinergic Signaling

Osteocytes actively regulate bone remodeling through purinergic signaling, in which extracellular ATP (eATP) acts as a key messenger. Under mechanical loading or stress, osteocytes can release ATP into the microenvironment via Cx43 hemichannels, a process linked to mechanosensitive inputs such as Piezo1, providing a direct route by which mechanical cues drive eATP signaling [7,95].

This eATP signal is then transduced through purinergic receptors on surrounding cells, with P2X7 standing out as a clinically relevant target because it is directly activated by eATP and implicated in inflammation and bone remodeling [96]. In osteoclast biology specifically, accumulating evidence connects P2X7 signaling to osteoclast differentiation and resorption; P2X7 knockdown suppresses osteoclastogenesis via autophagy and Ca^2+^/calcineurin-associated pathways, supporting the concept that modulating P2X7 can blunt osteoclast function and limit osteolysis in disease [58,97]. Since osteoclast-driven osteolysis also sustains tumor–bone feedback loops, dampening osteoclast activity via eATP→P2 receptor signaling is positioned as a translational strategy to protect bone integrity in malignant settings [98].

Beyond bone resorption, eATP signaling can also influence tumor behavior in the bone niche. ATP has been shown to inhibit breast cancer cell migration and bone metastasis through purinergic receptor signaling (via P2Y11 in that model), underscoring that extracellular purines can directly modulate metastatic traits rather than acting only as generic “stress” signals [32]. In parallel, P2X7 biology has been linked to cancer progression and bone metastasis in prostate cancer contexts, further supporting purinergic receptors as actionable nodes at the tumor–bone interface [99]. Current and emerging therapeutic strategies targeting osteocyte-regulated pathways, including their mechanisms, evidence level, and major limitations, are summarized in Table 2.

Together, these findings support a focused, testable axis: leverage osteocyte Cx43 hemichannel–mediated eATP release to engage P2X pathways, particularly pharmacologic modulation of P2X7, to inhibit osteoclast differentiation/resorption and, in turn, dampen tumor-promoting dynamics in bone [7,58,95,96,97,98].

### 6.6. Senolytics/Senomorphics

Evidence is increasingly linking osteocyte senescence to the biology of metastatic bone disease. In breast cancer bone metastasis, single-cell and functional data indicate osteocytes can undergo premature senescence associated with altered signaling and bone destruction, implicating senescent osteocytes as modifiable niche regulators rather than passive bystanders [36,54]. Senescence may drive niche “permissiveness” via the SASP—pro-inflammatory and tissue-remodeling factors that can promote osteoclast activity, suppress osteoblast function, and alter immune regulation [36,54,100].

Translationally, this motivates senescence-targeting strategies to remodel the metastatic niche. Senolytics that eliminate senescent cells have proof-of-concept in marrow-associated microenvironments; navitoclax (ABT-263) has been used in preclinical trials to reduce senescent niche components, supporting pharmacologic tractability [101]. Human data also support the clinical feasibility of intermittent senolytic regimens with bone-metabolism outcomes [102]. In parallel, senomorphic/SASP-directed approaches aim to blunt harmful secretory outputs; a cited example is liposome-encapsulated fisetin reducing SASP cytokines (including IL-6 and IL-8) without clear senolysis, supporting the broader rationale for SASP-targeted agents (e.g., IL-6–axis interventions) as a niche-modifying strategy [103].

Finally, the observation of senescent stromal populations in pre-metastatic bone marrow in advanced breast cancer suggests senescence may contribute early to a permissive environment, strengthening the case for testing senolytic (e.g., navitoclax) and SASP-suppressing approaches in dedicated bone metastasis models to determine whether targeting osteocyte/stromal senescence can reduce niche permissiveness and metastatic progression [36,54,104].

**Table 2:** Therapeutic strategies targeting osteocytes: mechanism, evidence level, and risks.

Strategy	Mechanism	Evidence Level	Main Risk/Limitation	References
Denosumab	Inhibits RANKL-dependent osteoclastogenesis in osteocyte-regulated bone remodeling	Established clinical use	Hypocalcemia, osteonecrosis of the jaw, and unclear effects on osteocyte-driven dormancy/persistence	[65,67,69]
Anti-sclerostin therapy	Relieves SOST-mediated Wnt inhibition and promotes bone formation	Clinical bone-anabolic use; metastasis evidence remains preclinical/mixed	Context-dependent effects on tumor behavior and possible concern in Wnt-responsive cancers	[32,75,77]
DKK1 inhibition	Restores Wnt signaling and osteoblast activity	Early translational rationale	Uncertain effect on overall tumor behavior and integration with standard therapy	[79,81]
Piezo1 agonism	Enhances osteocyte mechanosensation and anti-resorptive/anabolic signaling	Preclinical proof-of-concept	No established clinical agents; safety and dosing remain unresolved	[35,83]
LMHF vibration	Activates osteocyte mechanotransduction	Preclinical proof-of-concept	Standardization of dose, duration, and patient selection is needed	[36,85]
Cx43 activation	Increases ATP/PGE2 release, supports osteocyte survival, and suppresses osteoclast activity	Preclinical proof-of-concept	No human clinical data; delivery and specificity require optimization	[42,93]
PLR/acidification targeting	Inhibits osteocyte-mediated matrix remodeling and acidification	Conceptual to preclinical rationale	Potential systemic toxicity; targeted delivery is likely needed	[58,98]
Purinergic modulation	Targets ATP-related signaling affecting osteoclast and tumor behavior	Emerging rationale	Optimal targets and translational strategy remain unclear	[41,101,104]

LMHF, low-magnitude, high-frequency; PLR, perilacunar/canalicular remodeling; ATP, adenosine triphosphate; PGE2, prostaglandin E2.

### 6.7. EV-Directed Strategies

#### 6.7.1. EV Biogenesis and Uptake Inhibitors

Extracellular vesicles (EVs), particularly exosomes, are critical mediators of tumor–bone crosstalk, enabling cancer cells to condition the bone microenvironment for metastasis. Inhibiting EV biogenesis and uptake offers possible therapeutic strategies to disrupt this communication and reduce metastatic progression [105]. Tumor EV biogenesis depends on regulated pathways, such as those involving neutral sphingomyelinase 2 (nSMase2) and Rab proteins, which are viable targets for pharmacological inhibition. nSMase2 inhibitors, for instance, show potential to reduce EV secretion and weaken tumor-driven remodeling of the bone niche, offering a more targeted alternative to nonspecific interventions [106,107,108]. Additionally, blocking EV uptake via mechanisms such as micropinocytosis could prevent functional delivery of EV cargo to bone-resident and stromal cells, thereby interrupting pro-metastatic signaling cascades [109,110].

#### 6.7.2. Engineered Exosomes with Bone Tropism for Drug Delivery

Beyond EV inhibition, engineered exosomes with bone tropism offer an early proof-of-concept approach for therapeutic delivery. Unlike tumor-derived EVs, non-bone-derived engineered vesicles may offer superior targeting capabilities, enabling precise delivery of therapeutics to osteocytes and other bone-resident cells. This strategy leverages the natural ability of exosomes to transport molecular cargo, while modifying their tropism to enhance specificity toward skeletal tissues [105,111]. Such vesicles could be used to deliver EV inhibitors or other drugs directly to the bone microenvironment, reducing systemic toxicity and maximizing therapeutic efficacy.

### 6.8. Systemic Modulators Impacting Osteocytes

Accumulating evidence highlights the role of β2-adrenergic receptor (β2AR) signaling in promoting bone metastasis by modulating host bone cells, including osteoclasts and stromal cells. Sympathetic nervous system (SNS) activation and downstream adrenergic signaling have been shown to enhance metastatic progression by reshaping the bone microenvironment, supporting tumor colonization, and altering bone remodeling dynamics [112,113]. In particular, β2AR activation has been associated with increased osteoclast activity, directly linking adrenergic tone to pro-metastatic bone remodeling processes [113].

Pharmacological β-blockade, a well-established approach to tamp down sympathetic tone, offers a promising strategy to disrupt these tumor-promoting pathways. β-blockers could mitigate β2AR-driven enhancement of bone metastasis by modulating both tumor behavior and the bone microenvironment. Preclinical and mechanistic studies support the potential of β-blockers to influence immune remodeling, stromal conditioning, and bone niche permissiveness, providing a strong translational rationale for their use in bone-metastatic disease [112,113,114,115,116].

While preclinical evidence is compelling, prospective clinical data are needed to validate the efficacy of β-blockers in mitigating metastasis-associated bone disease. Observational studies have suggested potential benefits in certain cancer settings, but robust trials are necessary to confirm their utility and identify specific patient populations most likely to benefit [112,117,118].

Adrenergic signaling may also intersect with osteocyte biology, as osteocytic cells have been reported to express β-adrenergic receptors and to alter RANKL/OPG signaling in response to β-adrenergic stimulation. However, direct evidence linking osteocytic β-adrenergic signaling to metastatic bone progression remains limited, so this pathway is best viewed as a contextual rather than a core osteocyte-targeted axis.

Overall, targeting β2AR signaling through β-blockade represents a rational systemic strategy to address the adrenergic contributions to bone metastasis, with the potential to reduce metastatic progression by interfering with tumor–bone cell interactions and remodeling dynamics [112,113].

## 7. Biomarkers and Imaging Readouts of Osteocyte Activity in Metastasis

Osteocytes are emerging as key regulators of bone remodeling and metastatic bone disease, making their secretory outputs and structural changes clinically relevant for monitoring skeletal metastases. Serum biomarkers such as sclerostin (SOST) and DKK1 represent mechanistically significant readouts of osteocyte activity. Sclerostin, a Wnt signaling antagonist, reflects osteocyte mechanotransduction and remodeling dynamics, whereas DKK1 has been implicated in Wnt pathway dysregulation in osteolytic metastases, particularly in cancers such as NSCLC [38,119,120]. These biomarkers, along with bone turnover markers such as NTX and bone-specific alkaline phosphatase (BAP), support dynamic monitoring of bone remodeling and metastatic burden across cancer types [121,122,123].

Imaging also plays a pivotal role in capturing osteocyte-related structural changes. High-resolution microCT enables visualization of lacunar-canalicular network (LCN) integrity and perilacunar remodeling (PLR), with altered lacunar density and morphology serving as proxies for osteocyte network disruption in metastatic bone [124,125]. These imaging-derived metrics complement serum biomarker data by providing lesion-specific insights into osteocyte-driven remodeling.

Mechanistic studies further highlight osteocyte signaling pathways, such as ATP and prostaglandin E_2_ (PGE_2_) release mediated by connexin 43 (Cx43) activation, as contributors to osteocyte remodeling and metastatic niche evolution. Additionally, single-cell and spatial profiling in human metastatic bone lesions have revealed osteocyte senescence, linking cellular aging to disrupted bone remodeling and metastatic progression [10,38,92,126].

By combining serum biomarkers (e.g., sclerostin, DKK1), turnover markers (e.g., NTX, BAP), imaging proxies for LCN integrity, and mechanistic insights into osteocyte signaling and senescence, a comprehensive framework can be developed for diagnosing, stratifying, and monitoring metastatic bone disease [92,119,120,124,125]. These integrated approaches improve upon traditional imaging alone, offering a multi-layered strategy to better understand and manage tumor–bone interactions.

## 8. Conflicting Data and Nuances

### 8.1. Tumor Context 

Anti-sclerostin drugs augment bone mass by disinhibiting canonical Wnt/β-catenin signaling in osteoblast-lineage cells; nevertheless, Wnt also plays a crucial role in promoting tumor growth and stemness in various malignancies. The existing findings indicate a context-dependent “double-edged” phenomenon in skeletal metastasis that correlates closely with the tumor’s responsiveness to Wnt ligands.

A study on breast cancer in mice revealed that sclerostin antibody or Sost deficiency significantly enhanced bone metastases of MDA MB 231 cells, which exhibit a strong response to Wnt3a, but did not affect Wnt-responsive cell lines. Anti-sclerostin elevated the Wnt target gene *AXIN2* and nuclear β-catenin in bone-colonized tumor cells, signifying direct stimulation of canonical Wnt signaling in metastases [127]. Anti-sclerostin promoted tumor sphere development, indicative of a stem-like characteristic, without augmenting two-dimensional proliferation or migration, implying the expansion of a stem-like subpopulation rather than overall growth [127]. Anti-sclerostin elevated the number of osteoclasts and their precursors in metastatic bone, perhaps exacerbating the “vicious cycle” of osteolytic metastasis [127]. Inhibition of sclerostin enhances systemic bone development; nevertheless, in Wnt ligand-responsive breast cancer cells located in bone, the same Wnt activation may promote tumor stemness and increase metastatic burden.

In multiple myeloma, where Wnt activation is important in pathogenesis, many models demonstrate that sclerostin inhibition restores bone integrity without promoting tumor development. Neutralizing sclerostin reinstates osteoblast functionality, repairs lytic lesions, and enhances bone mass; however does not promote multiple myeloma tumor proliferation in several preclinical models or ex vivo human bone organ cultures [128,129]. In combined therapy, the sclerostin antibody did not diminish the efficacy of a potent anti-multiple myeloma Notch inhibitor, and tumor burden was only influenced by the anti-cancer medication, rather than by the co-administration of the sclerostin antibody [128]. A restricted retrospective cohort of multiple myeloma patients in remission, treated with Scl ab for bone repair, demonstrated enhanced bone mass without any recorded disease relapse during a brief follow-up period, but the sample size and length were constrained [128]. Narrative and mechanistic assessments consistently indicate that in multiple myeloma and several breast cancer models, sclerostin inhibition either exerts no effect or diminishes tumor growth in bone, concurrently enhancing bone strength [129,130].

The primary differentiator in the breast cancer study is the tumor’s susceptibility to Wnt ligands. Wnt-responsive metastases (e.g., MDA MB 231) exhibited significant responsiveness to Wnt3a in TOPFLASH/AXIN2 tests, wherein sclerostin inhibition resulted in enhanced beta-catenin signaling inside the tumor, augmented tumor sphere development, and an increase in metastases [127]. Conversely, Wnt unresponsive or weakly responsive metastases exhibited negligible Wnt3a response in vitro, and anti-sclerostin did not enhance bone metastases of these lines [127].

In multiple myeloma, tumor cells may respond to Wnt under certain conditions; however, sclerostin inhibition primarily reactivates osteoblasts and mitigates Wnt antagonism in the niche, without triggering a proliferative Wnt transcriptional program in multiple myeloma cells [128]. Consequently, the identical systemic intervention may be mostly osteoanabolic and anti-resorptive, with Wnt activation restricted to the osteoblast/osteocyte compartment, encompassing PDGFR–M CSF regulation and diminished osteoclastogenesis [131]. Conversely, in instances when tumor cells are receptive to Wnt ligands and exist within a Wnt-abundant bone microenvironment, the intervention may also stimulate beta-catenin in metastases, hence augmenting stemness and metastatic load [127].

In general, anti-sclerostin therapy seems to be safer in contexts where tumor cells are either Wnt-unresponsive or absent (such as multiple myeloma in deep remission or non-malignant osteoporosis); nonetheless, it may pose a potential metastatic risk in the presence or suspicion of Wnt ligand-sensitive tumors with a propensity for bone involvement.

### 8.2. Cell Stage and Load Regime 

Piezo1 is expressed throughout the osteoblast lineage, but its prevalence and functional significance vary with maturation: it facilitates osteocyte differentiation and dendrite development during the late osteoblast-to-osteocyte transition, whereas Piezo1 in fully mature osteocytes is less essential for adaptive loading responses. The absence of Piezo1 in late osteoblasts and osteocytes impairs osteocyte differentiation and diminishes both the number and length of dendrites, underscoring the essential function of Piezo1 in the maturation process [132].

Osteoblast-lineage cells express piezo1, which facilitates mechanosensitive transcriptional responses in whole-animal models and cultured osteocyte-lineage cells when bone mass is decreased by lineage deletion [132]. Despite being reduced, Piezo1 is functionally retained in mature osteocytes, as the targeted deletion in these cells modifies steady-state bone mass and microstructure, indicating its expression and physiological significance in mature cells [133]. Intermittent stimulation of downstream effectors (YAP) or systemic overexpression of CCN1/2 mitigated dendritic and bone abnormalities resulting from Piezo1 deficiency, hence endorsing a differentiation-stage role facilitated by Piezo1-mediated signaling [132].

Pharmacologic Piezo1 (Yoda1) activation can restore glucocorticoid-suppressed differentiation and mechanosensitivity in periosteal/osteoprogenitor contexts and replicate flow-induced gene responses in cultured osteocyte-lineage cells, which is consistent with its function in promoting or restoring differentiation-linked mechanisms [132,134]. In models of glucocorticoid-induced osteoporosis, the expression of Piezo1 is modulated by HES1, and the activation of Piezo1 promotes the phosphorylation of CaMKII and Akt, thereby reinstating mechanosensitive responses and lacunocanalicular integrity [134].

Piezo1-induced Ca^2+^ signals activate Cx43 hemichannels through the PI3K-Akt pathway and initiate ATP–P2X signaling and Panx1 to maintain intracellular Ca^2+^ responses to mechanical stress in osteocytes [135]. The conditional deletion of Piezo1 from the osteoblast/osteocyte lineage (Dmp1-Cre) disrupts the bone anabolic response to mechanical loading, while deletion limited to mature osteocytes (Sost-Cre) reduces bone mass without inhibiting the skeletal loading response. This suggests that mechanosensory dependence is more pronounced in earlier and late-differentiating lineage cells than in fully mature osteocytes [133]. In MLO-Y4 cells, fluid shear enhances Piezo1 and modifies OPG/RANKL expression through NOTCH3, suggesting that Piezo1-dependent mechanosensing affects osteoclastogenic signaling in osteocyte-like cells [136]. One model showed unanticipated Wnt/β-catenin activation when Piezo1 was lost in the lineage, indicating a possible interaction between Piezo1-driven mechanotransduction and the normal bone anabolic pathways during differentiation [132].

Extensive deletions in osteoblast/osteocyte lineages (Dmp1 Cre, Prx1 Cre, Runx2 Cre) result in osteoporosis due to modified osteoblast–osteoclast interactions and heightened resorption, highlighting Piezo1 as a pivotal mechanosensor in the skeleton [135,137,138,139,140,141,142]. Although these models demonstrate significant alterations in the bone microenvironment, metastasis is not yet explicitly studied. Microfluidic and in vitro studies demonstrate that Yoda1 or Piezo1-mediated osteocyte activation under low magnitude high frequency vibration can: Reduce MDA MB 231 breast cancer cell migration through osteoclast-dependent pathways [27]; increase nuclear YAP in osteocytes and inhibit osteoclastogenesis; decrease breast cancer cell extravasation into bone mimetic settings; Piezo1 activation amplifies this inhibitory impact [26]. These findings suggest that osteocytes’ functioning of Piezo1 contributes to the formation of a microenvironment that inhibits the initial stages of bone metastasis [26,27]. No in vivo models have directly tested bone metastasis burden in Piezo1-deficient mature osteocyte backgrounds [26,27]. It is unknown whether osteocyte-specific Piezo1 loss alone, without systemic bone loss, is sufficient to alter metastatic seeding, dormancy, or outgrowth.

### 8.3. Tumor Type Heterogeneity in Engaging Osteocytes

Single-cell profiling among various cancers reveals a minimum of three “ecosystem archetypes” of bone metastasis, characterized by macrophages/osteoclasts, Treg/exhausted T cells, or monocytes, with only a modest correlation to the tissue of origin, highlighting both convergent and divergent pathways to bone colonization [143].

Bone metastases from breast cancer are primarily osteolytic and characterized by a macrophage/osteoclast (Mφ OC) archetype, triggered by the typical “vicious cycle”: tumor-derived substances promote osteoclastogenesis and bone resorption, which in turn releases growth factors that further boost tumor proliferation. Single-cell findings indicate that breast bone lesions predominantly cluster within the Mφ OC archetype, characterized by robust osteoclast programs and estrogen-responsive macrophages [143].

Breast metastases exhibit microenvironmental variability across different sites: bone lesions demonstrate increased neutrophil infiltration relative to primary tumors, while lymph node metastases exhibit reduced macrophage presence [144,145]. In scRNA sequencing of primary tumors, lymph nodes, brain, and bone metastases, bone lesions are distinguished by elevated levels of proliferative cancer-associated fibroblasts (pCAFs) interacting with malignant epithelial cells, which are associated with an unfavorable prognosis [144]. Tumor cells co-cultured with cancer-associated fibroblasts (CAFs) exhibit increased expression of IL-6 and IL-8, hence augmenting invasiveness and angiogenesis [144]. Marrow adipocytes facilitate breast and melanoma bone metastases by supplying lipids and pro-inflammatory mediators (e.g., PPARγ, IL-1β, IL-6, FABP4, CXCL1/2) that enhance tumor proliferation and osteolysis [146].

Prostate cancer bone metastases are often osteoblastic, though frequently mixed; there is considerable molecular and stromal heterogeneity. Transcriptomic profiling identifies three molecular subtypes (MetA–C); MetB exhibits elevated proliferation, diminished PSA levels, and a suboptimal response to androgen restriction therapy [147]. Histologically, the majority of metastases contain several epithelial subclones exhibiting diverse morphologies and are situated within a heterogeneous stroma characterized by varying bone content, vascular density, and fibroblast markers [147]. Two stromal subtypes are identified: one characterized by high bone density, reduced vascularity, and a favorable prognosis, and another marked by increased proliferation, greater vascularity, and poorer results [147].

Chemokine signaling influenced by the microenvironment modulates prostate bone disease. Prostate cancer cells that metastasize to bone stimulate mesenchymal stem cells (MSCs) to produce CXCL8 and its murine counterpart Cxcl1; MSC-derived Cxcl1 inhibits osteoblast development, fosters a pro-tumoral immunological environment characterized by immature neutrophils, and facilitates tumor proliferation [148]. The genetic deletion of Cxcl1 in mesenchymal stem cells (MSCs) promotes osteogenesis, modifies immunological composition (resulting in a reduction in immature neutrophils and an increase in monocytes), and significantly inhibits prostate tumor growth in bone [148]. Previous research also associates CXCL8 family chemokines with osteolytic bone metastases in breast cancer and multiple myeloma, where they promote osteoclastogenesis and tumor viability [148].

Heterogeneity in kinase signaling further differentiates bone from other metastatic locations. In PTEN-deficient mice, bone metastases, unlike those in the lung or liver, demonstrate heightened PI3K/mTOR signaling and c MET expression; suppression of c MET selectively disrupts bone lesions [145]. EpCAM high epithelial cells exhibiting elevated PI3K/mTOR activity coexist with EpCAM low mesenchymal cells displaying reduced kinase activity, resulting in varied susceptibility to PI3K/mTOR inhibitors [145].

Multiple myeloma results in characteristic widespread osteolytic bone disease accompanied by significant skeletal consequences. Although the included publications exhibit limited representation of precise molecular variability, numerous consistent features are evident throughout preclinical models. Multiple myeloma bone disease has clinical endpoints analogous to those of solid bone metastases, including fractures, pain, and diminished quality of life. It is often replicated in conjunction with breast and prostate bone metastasis using intratibial systems that replicate osteolytic lesions and pain behaviors [149,150].

The activation of osteoclasts induced by chemokines is fundamental. CXCL8 is increased in bone stromal cells in multiple myeloma and facilitates both myeloma survival and tumor-associated osteolysis, mirroring the activities of the CXCL8 family in osteolytic breast cancer and in the MSC-mediated regulation of prostate bone metastases [148]. Increased osteocyte apoptosis has been observed in multiple myeloma lytic lesions, signifying a significant role of osteocyte death in bone degradation, a phenomenon that is now also mechanistically elucidated in melanoma [151]. Bone marrow adipocytes seem to significantly facilitate multiple myeloma and prostate bone disease, followed by breast cancer and melanoma, via metabolic and cytokine interactions; nevertheless, clinical data are limited [146].

Bone metastases from melanoma are less prevalent than visceral or cerebral metastases but are associated with significant morbidity due to osteolytic lesions and skeletal-related complications. Lesions are predominantly osteolytic and mediated by osteoclasts via both RANKL-dependent and independent mechanisms [152]. Tumor-associated macrophages, myeloid-derived suppressor cells, regulatory T cells, and cancer-associated fibroblasts establish an immunosuppressive tumor microenvironment that facilitates osteoclastogenesis through cytokines such as IL-6 and CXCL8, which activate the JAK/STAT3, NF-κB, and MAPK pathways in osteoclast precursors and melanoma cells [152]. In addition to immune cells, systemic variables influence melanoma formation in bone. The depletion of the gut microbiome due to broad-spectrum antibiotics accelerates intraosseous melanoma proliferation and osteolysis by hindering the expansion of NK and Th1 cells in the intestine and their CXCR3/CXCL9-mediated migration to tumor-infiltrated bone marrow; obstructing lymphocyte egress or CXCR3/CXCL9 signaling replicates this acceleration [153].

Recent elucidations at the level of bone resident cells have identified two osteocyte-centered processes that underscore distinctive melanoma-bone interactions: ferroptotic osteocyte death and CXCL5-mediated tumor support. Melanoma bone metastases induce significant osteocyte mortality mostly through ferroptosis, rather than apoptosis or necroptosis. RNA sequencing of osteocyte-enriched calcified bone demonstrates an enrichment of ferroptosis pathways and signatures associated with “response to iron ion”, characterized by the downregulation of GPX4 and upregulation of HMOX1 [151]. Mechanistically, HIF1α functions upstream of HMOX1, promoting excessive autophagy-mediated ferritin breakdown (ferritinophagy), resulting in iron overload and lipid peroxidation, ultimately leading to osteocyte ferroptosis [151]. The suggested loss of osteocytes is believed to contribute to further bone loss and remodeling in melanoma tumors [151].

A separate and complementary route entails osteocyte-derived CXCL5 as a prometastatic signal exclusively induced by melanoma cells. Through RNA sequencing and chemokine analysis, osteocytes exposed to melanoma cells significantly upregulate and release CXCL5 [44]. This activation is not merely basal chemokine production but rather a tumor-conditioned response that reprograms osteocytes into active facilitators of metastatic development. 

Osteocyte-derived CXCL5 functionally promotes melanoma cell motility and invasion in vitro through the CXCR2 receptor on melanoma cells [44]. Silencing of CXCR2 in melanoma significantly diminishes their migratory response to osteocytes, indicating that the communication between osteocytes and tumors occurs via a CXCL5–CXCR2 axis. In vivo, melanoma cells expressing lower levels of CXCR2 produce fewer bone metastases and result in reduced bone loss in murine models [44].

Importantly, simultaneous targeting of CXCL5 in osteocytes and CXCR2 in melanoma cells nearly entirely halts melanoma growth in bone in vivo. This bilateral necessity emphasizes CXCL5 induction as a point of reciprocal interaction rather than a unidirectional tumor-intrinsic program [44]. In melanoma, CXC chemokines such as CXCL1, CXCL5, and CXCL8 are overexpressed in skin tumors and linked with prognosis and inflammation; however, the unexpected result is that osteocytes, rather than melanoma cells alone, are the primary source of CXCL5 in the metastatic niche [44,154].

## 9. Knowledge Gaps and Future Experiments

### 9.1. Causal Mapping of LCN Topology Changes vs. DTC Fate

In murine osteotropic breast and prostate cancer, cortical regions with tumors exhibit enlarged lacunae, compromised canalicular connection, and localized alterations in vascular canal density in comparison to controls, signifying significant LCN remodeling at metastatic sites [3,155]. The structural alterations are associated with osteolytic and osteosclerotic areas, as well as initial declines in bone mechanical competence, indicating that tumor–osteocyte interactions modify the LCN and bone strain perception [3,155]. Osteocyte network density and structure fluctuate with mineral content and across cortical areas in robust bone, influencing mechanosignaling capability [156,157].

Numerous studies indicate that signals originating from osteocytes, particularly under mechanical pressure, play a role in either sustaining or disrupting tumor dormancy. The transfer of osteocyte mitochondria can activate STING-dependent antitumor immunity, hence restricting metastatic development in bone [158].

Reviews highlight osteocytes as essential regulators of metastatic niche signaling (cytokines, EVs, mechanotransduction), although they fail to specify certain LCN network features (degree, path length, clustering) as factors influencing DTC dormancy versus outgrowth [3,10,159,160].

Recent results indicate that osteocytes and their mechanosensitive signaling, partially reliant on the LCN, can facilitate tumor dormancy or inhibit metastatic advancement in bone. Nonetheless, no research currently establishes that the spatial architecture of the LCN serves as a causal regulator that determines DTC dormancy vs. metastatic expansion, rather than merely acting as a consequence or modification of larger osteocyte-tumor interactions.

### 9.2. In Vivo Dissection of Osteocyte EV Cargo on Tumor Dormancy/Full Outgrowth

The sole directly pertinent in vivo research linking osteocyte-derived sEVs, miRNA cargo, and cancer cell activity is the recent study on NSCLC bone metastases conducted by Xie et al. [161]. Other sEV–miRNA studies elucidate pathways of miRNA-mediated tumor suppression or bone remodeling; however, they are either not obtained from osteocytes or not conducted in bone metastasis models [162,163,164,165].

In a model of bone metastases in non-small cell lung cancer (NSCLC), osteocytes detecting mechanical stimulation (by tibial loading or treadmill exercise) released small extracellular vesicles (sEVs) enriched in tumor suppressor microRNAs, particularly miR 99b 3p [161]. Gain and loss of function experiments identified miR 99b 3p as the principal mediator, directly targeting the E3 ligase MDM2 through its 3′ UTR, thereby functionally alleviating p53 from MDM2-mediated repression and restricting NSCLC proliferation [161].

Although not based on bone osteocytes, numerous in vivo investigations corroborate the overarching notion that sEV-supplied tumor suppressor microRNAs can inhibit metastatic proliferation. Intraperitoneal delivery of miR–29 b–loaded bone marrow MSC sEVs inhibited peritoneal metastases of gastric cancer by counteracting TGFβ-induced mesothelial epithelial–mesenchymal transition-like alterations and diminishing tumor adherence and colonization [165]. The fate of disseminated tumor cells within the bone microenvironment is governed by microenvironmental stress, TGFβ–p38–mediated dormancy programs [166], immune surveillance [167,168], and the nature of the pre-metastatic niche [169,170,171].

### 9.3. Trials That Embed Osteocyte-Centric Endpoints (SOST/DKK1 Dynamics; Cx43-HC Activity; Senescence Markers) into Standard Anti-Resorptive Regimens

Osteocytes modulate bone formation and resorption through Wnt antagonists (sclerostin, DKK1) and connexin 43 hemichannels (Cx43 HC). These signals are more closely associated with alterations in bone microarchitecture than traditional resorption indicators such as CTX, which just indicate collagen degradation.

Sclerostin and DKK1 are primarily released by osteocytes, directly inhibiting Wnt/β-catenin signaling, which suppresses osteoblast development and promotes osteoblast death, therefore leading to increased osteoclastogenesis and bone resorption [172,173,174]. Increased sclerostin has been associated with diminished bone formation, enhanced resorption, and decreased bone mineral density in osteoporosis, chronic kidney disease, metabolic bone disorders, Gaucher disease, and diabetes-related bone fragility [172,173,174,175,176,177]. CTX indicates present osteoclast activity; nevertheless, it fails to account for osteocyte-mediated alterations in remodeling set point or cortical porosity, which are essential factors influencing bone quality.

In multiple myeloma, sclerostin and DKK1 levels correlated with bone lesion burden and therapeutic response; reductions during treatment coincided with enhancements in myeloma bone disease, indicating their utility for monitoring treatment-induced alterations in bone integrity, particularly when CTX is influenced by disease activity [178]. In Gaucher disease, increased sclerostin is significantly correlated with bone pain, marrow infiltration, Erlenmeyer deformity, and osteopenia/osteoporosis, as the usual sclerostin–DKK1 equilibrium is disrupted with declining bone mineral density, thereby directly connecting these osteocyte markers to structural pathology and symptoms [177]. A case–control study on osteoporosis indicated significantly elevated serum sclerostin levels in osteoporotic participants compared to controls, demonstrating strong diagnostic sensitivity and specificity for osteoporosis status [179].

In postmenopausal osteoporosis treated with romosozumab, CTX decreases and BMD increases; nonetheless, Dkk1 progressively rises and is inversely correlated with P1NP, reflecting the diminished anabolic response despite ongoing CTX suppression [180]. DKK1 effectively encapsulates compensatory osteocyte responses and forecasts the decline in bone-forming ability that CTX fails to predict. Preclinical and clinical studies targeting Wnt demonstrate that anti-sclerostin therapy enhances bone mineral density (BMD) and reduces fracture incidence, while also altering both C-terminal telopeptide (CTX) and formation indicators; the Wnt inhibitors are pivotal in this mechanism [174,181,182]. Assessing sclerostin/DKK1 in these trials directly evaluates target engagement and aids in interpreting paradoxical changes in CTX levels.

Sclerostin and DKK1 serve as upstream regulators of cortical porosity, trabecular thickness, and turnover equilibrium; their levels closely correlate with variations in bone volume and structure across various disorders and therapies [172,175,176,177,178,182]. CTX is unable to differentiate between high turnover with maintained structure and high turnover accompanied by significant microarchitectural degradation. Sclerostin is influenced by mechanical stress, diabetes, chronic kidney disease, inflammatory conditions, and malignant bone disorders, positioning it as a systemic integrator of variables that compromise bone quality beyond mere resorption [172,173,174,177,183]. Data from CKD biopsies and transplant bone histomorphometry directly associate sclerostin/DKK1 with turnover category and bone volume, which are the gold standards for assessing bone quality, whereas CTX demonstrates little predictive capability at the individual histological level [172,175].

### 9.4. Mechanotherapy Dosing and Piezo-Safe Agonists Translation

Existing research suggests a mechanistically credible additive or synergistic interaction between mechanical dosage (vibration/loading) and Piezo1 agonists in promoting bone growth, along with nascent evidence on their impact on bone metastasis.

Moderate tibial loading combined with Yoda1 in approximately 50-week-old mice resulted in superior cortical and trabecular protection compared to either intervention alone, demonstrating enhanced cortical polar moment of inertia and a greater proportion of mice exhibiting beneficial structural modifications, indicative of at least additive effects. The identical loading profile combined with Yoda1 effectively reduced age-related bone loss more than individual treatments; however, it could not completely reverse severe damage generated by doxorubicin, suggesting that synergy has limitations under extreme cytotoxic stress [184]. In 22-month-old mice, tibial loading with an adjusted waveform that increases fluid flow while maintaining modest strain, in conjunction with Yoda2 injections, synergistically enhanced cortical parameters; in vivo Ca^2+^ imaging validated osteocyte Piezo1 activation by Yoda2 during loading [185]. Whole body vibration combined with Yoda1 in young mice approximately doubled bone growth compared to controls, while in mature mice, the combination (with a reduced duration of 15 min vibration) mitigated cortical polar moment loss, where individual treatments were predominantly ineffective, suggesting an age-dependent synergy [186].

Mechanistically, Piezo1 serves as a pivotal mechanostat in osteoblasts, osteocytes, and BMSCs: mechanical stress or vibration activates Piezo1, resulting in Ca^2+^ influx, thereby promoting osteoblast differentiation and inhibiting osteoclastogenesis [135,137,138,139,187]. Yoda1/Yoda2 or more recent agonists replicate or enhance these pathways and can repair osteoporosis caused by disuse, glucocorticoids, and age in preclinical animals [135,139,187,188,189]. Reviews explicitly indicate that Piezo1 agonists exhibit synergy with vibration and emphasize the necessity to adjust vibration frequency and intensity as a formal “dose” variable [187].

Certain studies indicate that moderate loading alone can safeguard against breast cancer-induced osteolysis, while Piezo1 activation in osteoclast lineage cells inhibits NFATc1 and osteoclastogenesis, even in models of inflammatory bone loss. This underscores a convergent pathway through which mechanical load and agonists may collaboratively mitigate resorption and the formation of metastatic niches [137,184,190].

Elevated frequencies (vibration, ultrasound) predominantly stimulate interstitial fluid movement and Piezo1 activation in osteocytes and osteoblasts, while moderate frequency dynamic loading (approximately 4 Hz) induces tissue-level strains alongside fluid flow; optimized low strain, high flow profiles appear especially efficacious when paired with agonists in aged bone [185,186]. Prolonged or excessive activation of Piezo1 (e.g., large systemic doses of Yoda1) can lead to cortical perforation and detrimental remodeling, especially in diseased or metastatic bone, indicating that safe synergy necessitates submaximal mechanical and chemical dosages [184,187]. Piezo1 in osteocytes, osteoblasts, bone marrow stem cells, macrophages, and pre-osteoclasts integrates mechanical and pharmacological stimuli to promote osteogenesis, diminish marrow adipogenesis, facilitate M2 macrophage polarization, and decrease osteoclastogenesis, thereby enhancing bone rigidity and potentially rendering it a less conducive environment for metastasis [137,184,187,188,189,191]. 

Optimal frequency/amplitude-drug dosing regimens and long-term safety require further dedicated studies, but current evidence supports a mechanistically grounded, context-dependent synergy between carefully dosed Piezo1 agonists and mechanotherapy for bone anabolism and partial protection against metastatic osteolysis.

Among these priorities, osteocyte-centric biomarkers and trial endpoints are the most immediately tractable because they can be embedded in ongoing bone metastasis studies using serum markers, imaging, and correlative tissue analyses. EV cargo mapping is likely the next most actionable area, particularly if lineage-resolved approaches can distinguish osteocyte-derived vesicles from other bone and tumor sources. By contrast, LCN topology is arguably of the highest mechanistic interest but remains the least clinically tractable in the near term because of technical constraints in resolving osteocyte network architecture in vivo in patients.

## 10. Clinical Perspective and Trial Design

### 10.1. Combine Denosumab with Cx43-HC Activators or Mechanotherapy

Denosumab effectively inhibits RANKL-mediated osteoclastogenesis and osteolysis, enhancing bone mass and diminishing the risk of skeletal-related events and bone metastases in high RANKL environments [192,193,194,195]. Independently, the activation of osteocytic Cx43 hemichannels (Cx43 HC), whether through pharmacological means or mechanical loading, promotes anabolic remodeling and anti-tumor signaling from osteocytes [7,30,33,196,197]. Current findings indicate robust molecular complementarity; however, there are no published trials that directly integrate denosumab with Cx43 HC activators or structured mechanotherapy.

Activation of Cx43 M2 in osteocytes diminishes TRAP^+^ osteoclasts at the endosteal surface, decreases bone resorption during unloading, and enhances cortical and trabecular mass in aged and unused bone [196]. Mechanical loading that activates Cx43 HCs diminishes SOST, amplifies β-catenin signaling, and redirects remodeling towards creation; conversely, when Cx43 HCs are compromised, loading unexpectedly elevates endosteal osteoclast activity [197]. Combined denosumab with Cx43 HC activation and mechanotherapy may offer dual anti-resorptive control through systemic RANKL inhibition and localized osteocyte-mediated suppression of osteoclastogenesis, achieved by reduced SOST levels, modified RANKL/OPG ratios, and maintained osteocyte survival [30,196,197].

Cx43 M2 modifies anti-tumor immunity by augmenting tumor-infiltrating effector T cells and diminishing regulatory T cells, transforming the bone-tumor niche from supportive to suppressive. Mechanical loading initiates the pathway Piezo1→Ca^2+^→PI3K–Akt→Cx43 HCs, resulting in prolonged Ca^2+^ and ATP signaling that promotes bone anabolism [7]. Reviews highlight the significance of Cx43 HCs and PGE_2_ in transducing mechanical stimuli into anabolic and potentially protective signals within bone [30,197]. In a skeleton treated with denosumab, where osteoclast-mediated “vicious cycle” signaling is diminished [192,193,194], the application of Cx43 HC activators or mechanotherapy may establish an osteocyte-driven microenvironment rich in ATP/PGE_2_ that is directly tumor-suppressive and immunostimulatory [7,30,33]. 

The discontinuation of denosumab leads to a significant resurgence in bone resorption, attributed to the accumulation of osteoclast precursors/osteomorphs and elevated RANKL levels, along with reduced OPG at bone surfaces and within osteocytes [16,198]. Activation of osteocytic Cx43 HCs diminishes osteocyte apoptosis and SOST during unloading, hence maintaining a more anabolic and less osteoclastogenic milieu [196,197]. Maintaining Cx43 HC activation and mechanical loading during transitions of denosumab may potentially mitigate rebound osteoclastogenesis; however, this has not been empirically verified. 

Cx43 signaling is contingent upon context: osteocytic Cx43 can facilitate osteoclastogenesis through JAK–STAT pathways and oxidative stress in wear particle osteolysis [199], while abnormal hemichannel opening may exacerbate inflammatory conditions [200]. Consequently, indiscriminate activation of Cx43 HCs may be detrimental in certain microenvironments. Denosumab’s immunological effects, mediated by RANKL on immune cells, connect with Cx43 HC-driven ATP and cytokine signaling.

### 10.2. If Contemplating Anti-Sclerostin, Stratify by Tumor Wnt Dependency

Anti-sclerostin antibodies (e.g., romosozumab, Scl ab) significantly stimulate canonical Wnt signaling in bone and serve as powerful osteoanabolics; however, Wnt is also a critical oncogenic pathway, and sclerostin plays context-dependent cardiovascular protective roles, resulting in a limited safety margin for patients with bone-tropic cancers [181,201,202,203,204,205]. In a breast cancer model, the inhibition of sclerostin specifically augmented bone metastases of Wnt-responsive MDA MB 231 cells, characterized by β-catenin accumulation in bone-colonized cells, improved tumorsphere formation indicative of stem-like properties, and an increase in osteoclasts and precursors inside the lesions [127]. This indicates that in Wnt ligand-responsive breast cancer, systemic suppression of sclerostin can expedite skeletal colonization and increase tumor burden in bone [127].

Multiple myeloma (MM) typically exhibits Wnt inhibition within the microenvironment, characterized by elevated levels of DKK1 and sclerostin. Neutralizing sclerostin reinstates osteoblasts and osteo CAR cells, and ameliorates lytic lesions without inducing multiple myeloma growth in various murine and ex vivo human bone models [75,206]. Preclinical models of multiple myeloma and breast cancer often demonstrate no effect or diminished tumor growth with sclerostin inhibition; however, the Hiraga study emphasizes that this is specific to cell lines and Wnt-dependent [70,75,127,206]. Reviews highlight that osteoanabolic Wnt stimulation in patients with a history of, or active, bone tropic malignancies poses a theoretical oncogenic risk, and existing preclinical and clinical data are inadequate for conclusive safety assessments [70,202,205].

In multiple myeloma models, the combination of anti-sclerostin monoclonal antibodies with zoledronic acid is suggested as a rational dual strategy to avert bone loss and multiple myeloma-related bone disease [75].

Notch serves as a crucial mediator of the interaction between multiple myeloma and the bone niche. The bone-targeted γ-secretase inhibitor (BT GSI) decreased multiple myeloma load and osteolysis more efficiently than systemic GSI, while circumventing gastrointestinal effects [206]. In multiple myeloma mice, the combination of Scl ab and BT GSI maintained the anti-tumor transcriptional program of BT GSI (upregulating apoptosis, downregulating proliferation and Notch targets), did not provoke pro-proliferative alterations in multiple myeloma cells, and facilitated bone healing without undermining tumor control [128,206]. 

In Wnt-driven metastasis, tumor cells utilize several converging pathways (Wnt, RANKL, Notch/Jagged1) to restructure the bone niche. The concurrent stimulation of bone formation (via anti-sclerostin) and targeted Notch inhibition within bone (e.g., BT GSI) may: restore normal osteoblast and osteo CAR cell function (bone-forming and immune-modulatory stromal cells) [128], inhibit tumor-promoting Notch signals and osteoclastogenesis [206,207,208], and diminish pro-metastatic Wnt effects by attenuating complementary niche signals.

Anti-sclerostin therapy for Wnt-dependent bone metastases poses significant risks of increasing skeletal colonization in Wnt-responsive malignancies and potential cardiovascular damage. Rational trial designs must focus on tumor Wnt biology, incorporate ongoing anti-resorptive or bone-targeted Notch suppression, and rigorously restrict exposure and eligibility to safeguard the bone niche without promoting tumor growth.

Given the context-dependent tumor effects of sclerostin blockade, any translational application should be interpreted in light of the Wnt-responsiveness framework summarized in Section 8.1.

### 10.3. Senolytics for Osteocyte SASP in Metastatic Niches

Therapy-induced senescence (TIS) resulting from chemotherapy, radiotherapy, PARP/CDK4/6 inhibitors, and certain antibodies generates a diverse population of senescent tumor and stromal cells that may exhibit acute immunostimulatory properties but are chronically driven by the senescence-associated secretory phenotype (SASP), leading to immunosuppression, resistance, relapse, and systemic toxicity [209,210,211,212,213,214,215]. “One-two punch” techniques combine a senescence-inducing primary drug with a secondary senolytic or SASP modulator to eliminate TIS cells and restructure the tumor microenvironment (TME) [209,210,212,213,216,217].

Senescent osteocytes in bone develop a pro-inflammatory, pro-osteoclastogenic secretory profile (SASP) that alters bone remodeling and the marrow immune niche. Crucial SASP elements, such as RANKL and IL-6, connect senescence to osteoclast activation, bone degradation, and an inflammatory, myeloid-biased microenvironment that could possibly promote metastasis. Senescent osteocytes significantly enhance osteoclastogenesis and bone resorption; the elimination of senescent cells using senolytics mitigates bone loss in metastatic models [36,54].

Osteocyte-induced senescence propagates to mesenchymal and myeloid progenitors, elevating SASP scores and systemic levels of TNF-α, IL-1β, and IL-6. The bone marrow transitions towards myelopoiesis, characterized by an increase in myeloid progenitors, neutrophils, and monocytes, while B-cell lymphopoiesis diminishes, resulting in a chronically inflamed, myeloid-dominant microenvironment [47].

TIS can initially enhance cGAS STING/type I IFN-mediated T cell recruitment and create “hot” tumor microenvironments (TMEs), but prolonged SASP leads to the predominance of tired CD8^+^ T cells, dysfunctional NK cells, and M2-like macrophages, thereby diminishing the efficacy of immune checkpoint inhibitors (ICIs) [210,214,218,219,220]. In murine models, the increase in senescent cells directly induces resistance to immune checkpoint inhibitors; pre-immunotherapy senolysis with navitoclax (ABT 263) reinstates CD8^+^ T cell infiltration, normalizes myeloid compartments, and enhances survival [221].

In various TIS situations, reliance on BCL xL represents a consistent vulnerability; BCL xL BH3 mimetics exhibit senolytic properties even when overall “mitochondrial priming” does not increase compared to proliferating cells [216]. Chronic SASP stimulates systemic senescent cell anti-apoptotic pathways (SCAPs) and induces secondary senescence in stromal and immunological cells; prolonged delays exacerbate off-tumor senolytic targets.

In “senescence-driven ICI resistance,” navitoclax administered prior to anti-PD-1/PD-L1 therapy eradicates senescent and senescence-conditioned myeloid cells, normalizes the tumor microenvironment, enhances CD8^+^ T cell proliferation, and reinstates ICI responsiveness [221]. Reviews indicate that pre-ICI senolysis can eliminate chronic SASP-mediated immunosuppressive niches (Tregs, MDSCs, M2 macrophages), thereby enhancing the eventual efficacy of checkpoint blockade. Inhibition of PARP and CDK4/6 in colorectal cancer elicits a pronounced tumor-intrinsic signature (TIS) and a type I interferon-rich senescence-associated secretory phenotype (SASP), which enhances CD8^+^ and natural killer (NK) cell infiltration while reducing granulocytic myeloid-derived suppressor cells (G MDSCs); the addition of αPD-L1 eradicates TIS cells and extends survival [219]. In this context, the “second punch” refers to immunotherapy rather than a traditional small-molecule senolytic, using immune-mediated elimination of senescent cells.

Most scheduling insights originate from preclinical models; comprehensive human pharmacodynamic data on senescence and SASP kinetics across various regimens are missing [209,210,211,214,216,217]. In vivo direct characterization of naïve and memory T cell sensitivity to clinical senolytics is limited; existing recommendations for safeguarding naïve T cells are predominantly inferential [214,218,222,223]. The heterogeneity of senescence (tumor, stromal, and immunological) indicates that senolysis at a single time point may be inadequate; dynamic, biomarker-guided approaches utilizing single-cell and spatial omics are recommended but have not yet become standard practice [209,211,214,218,224].

In the context of metastatic bone disease, the most compelling rationale for senescence-directed therapy is not senolysis in oncology at large, but the specific contribution of senescent osteocytes and adjacent stromal populations to a pro-resorptive, immunomodulatory bone niche. Thus, future work should prioritize bone-metastasis models that directly test whether osteocyte-targeted senolytic or senomorphic strategies reduce SASP output, preserve bone integrity, and limit metastatic outgrowth, rather than extrapolating broadly from non-skeletal tumor systems.

## 11. Conclusions

Osteocytes are no longer tenable as passive bystanders in metastatic bone disease; rather, the literature now supports their role as master integrators that translate mechanical strain, mineral demands, immune cues, and tumor-derived signals into coordinated remodeling outputs that can either constrain or enable metastatic success. Their embedded position within the lacunocanalicular network gives them unique access to sense altered loading and matrix microdamage, and to couple these inputs to endocrine/paracrine programs that shape osteoclastogenesis and osteoblast function (e.g., RANKL/OPG control, Wnt inhibition via sclerostin/DKK1), as well as to matrix turnover through perilacunar/canalicular remodeling (PLR) enzymes and acidification pathways [3,4,6,7,45]. In metastatic settings, osteocyte apoptosis and senescence-associated secretory programs amplify osteoclastogenic and inflammatory signaling, functionally linking osteocyte cell-fate decisions to osteolysis and tumor-supportive niche remodeling [1,31,36,51]. Mechanotransduction nodes such as Piezo1 and Cx43 hemichannels further position osteocytes as signal routers that convert mechanical inputs into ATP/PGE_2_-linked outputs with downstream effects on osteoclast activity, immune activation, and even direct tumor cell behavior [8,27,29,30,46,75]. Together, these advances shift the conceptual center of the “vicious cycle” from an osteoclast-centric loop to a broader osteocyte-governed systems framework in which osteocytes set the remodeling gain, sculpt niche architecture, and modulate immuno-skeletal tone across the metastatic cascade [1,2,3,61,62].

This reframing has immediate translational implications: osteocyte-directed therapies must be pursued with precision and context awareness—defined by tumor intrinsic signaling dependencies, lesion phenotype (osteolytic vs. osteoblastic/mixed), disease stage (seeding/dormancy/outgrowth), and host factors that alter mechanobiology and immune states [61,62,139]. Emerging modalities—mechanotherapy and Piezo1/Cx43 axis manipulation, purinergic signaling leverage, and senolytics/senomorphics targeting osteocyte/stromal SASP—are best viewed not as one-size-fits-all add-ons, but as niche-reprogramming tools whose benefit/risk profiles depend on dosing, timing, and microenvironmental state [75,77,78,79,83,84,91,92,93,94,95,96,98,99,127].

Ultimately, repositioning osteocytes as integrative regulators of mechanics, metabolism, immunity, and tumor ecology provides a coherent rationale for next-generation, osteocyte-informed trial designs that aim to preserve skeletal integrity while actively destabilizing the metastatic niche—by tailoring interventions to the biology of both the tumor and the osteocyte network that it attempts to co-opt [2,3,36,61,62,86,88,154,156].

## Figures and Tables

**Table 1:** Osteocyte signaling pathways and their effects on metastasis.

Pathway/Program	Key Mediators	Main Effect on Metastasis	References
RANKL/OPG	RANKL, OPG	Promotes osteoclastogenesis, osteolysis, and tumor outgrowth in bone	[12,18]
Wnt antagonism	SOST, DKK1	Suppresses osteoblast activity, favors a permissive niche, and contributes to osteolytic or mixed lesions	[15]
Mechanotransduction	Piezo1/2, Akt, YAP/TAZ	Physiologic loading restrains metastatic niche permissiveness, whereas impaired signaling may support progression	[8,13,37]
Cx43 hemichannel signaling	Cx43, ATP, PGE2	Supports bone homeostasis and may help maintain dormancy by limiting osteoclast activation	[12,39,41]
Apoptosis/senescence	HMGB1/RAGE, IL-6, TNF-α, SASP factors	Amplifies inflammation, osteoclastogenesis, and osteolytic progression	[4,43]
PLR	MMP13, CTSK, CA2	Enhances local matrix remodeling and supports the progression of established lesions	[5,14,58]
Extracellular vesicles	EVs carrying RANKL, OPG, SOST, miRNAs	May modulate bone remodeling and metastatic behavior, although evidence remains limited	[46]
Tumor-driven reprogramming	JAG1/NOTCH, CXCL5	Increases niche permissiveness, tumor cell survival, migration, and progression	[10,52]
Osteoimmune crosstalk	IL-6, TNF-α, RANKL, ATP/purinergic signaling	Can promote either anti-tumor immunity or immunosuppressive niche states depending on context	[53,56]

PLR, perilacunar/canalicular remodeling; EVs, extracellular vesicles; SASP, senescence-associated secretory phenotype; ATP, adenosine triphosphate; PGE2, prostaglandin E2.

## Data Availability

No new data were created or analyzed in this study. Data sharing is not applicable.

## Abbreviations

BAP	Bone-specific alkaline phosphatase
BIGH3/TGFBI	Transforming growth faction-β-induced protein
BMD	Bone mineral density
BMSC	Bone marrow mesenchymal stem cell
BT GSI	Bone targeted γ-secretase inhibitor
CaMKII	Ca^2+^/calmodulin-dependent protein kinase II
CAR cells	CXCL12-abundant reticular cells
CCN	Cyr61/CTGF/Nov family of proteins
CSF	Colony-stimulating factor
CTSK	Cathepsin K
CTX	C-telopeptide of type I collagen
Cx43 HC	Connexin 43 hemichannel
DKK1	Dickkopf-related protein 1
DTC	Disseminated tumor cell
EV	Extracellular vesicle
FABP	Fatty acid-binding protein
G-MDSC	Granulocytic myeloid-derived suppressor cell
GPX	Glutathione peroxidase
HES1	Hairy and enhancer of split 1
HIF1	Hypoxia-inducible factor 1
HMGB1	High mobility group box 1
HMOX1	Heme oxygenase 1
ICI	Immune checkpoint inhibitor
LCN	Lacunocanalicular network
LMHF	Low-magnitude high-frequency
MCP-1	Monocyte chemoattractant protein-1
NF-κB	Nuclear factor kappa B
NFATc1	Nuclear factor of activated T cells, cytoplasmic 1
NTX	N-telopeptide of type I collagen
OPG	Osteoprotegerin
P1NP	Procollagen type 1 N-terminal propeptide
PARP	Poly (ADP-ribose) polymerase
pCAF	Perivascular cancer-associated fibroblast
PD-L1	Programmed death-ligand 1
PDGFR-M	Platelet-derived growth factor receptor (Membrane)
PLR	Perilacunar remodeling
PPAR	Peroxisome proliferator-activated receptor
RAGE	Receptor for advanced glycation end products
RANKL	Receptor activator of nuclear factor kappa-Β ligand
SASP	Senescence-associated secsretory phenotype
scRNAseq	Single-cell RNA sequencing
SOST	Sclerostin
SREs	Skeletal-related events
TIS	Therapy-induced senescence
TME	Tumor microenvironment
TRAP	Tartrate-resistant acid phosphatase
Trm	Tissue-resident memory T cell
